# Rapid Genome Sequencing Shows Diagnostic Utility In Infants With Congenital Heart Defects

**DOI:** 10.21203/rs.3.rs-3976548/v1

**Published:** 2024-03-20

**Authors:** Matthew D. Durbin, Lindsey R. Helvaty, Alyx Posorske, Samuel Zhang, Manyan Huang, Ming Li, Daniel Abreu, Korre Fairman, Gabrielle C. Geddes, Benjamin M. Helm, Benjamin J. Landis, Alexis McEntire, Dana K. Mitchell, Stephanie M. Ware

**Affiliations:** a Indiana University School of Medicine, Indianapolis, IN; b Indiana University Bloomington School of Public Health, Bloomington, IN; c Washington University, St. Louis, MO; d Herman B Wells Center for Pediatric Research, Indianapolis, IN

## Abstract

Congenital heart disease (CHD) is the most common birth defect and a leading cause of infant mortality. CHD often has a genetic etiology and recent studies demonstrate utility in genetic testing. In clinical practice, decisions around genetic testing choices continue to evolve, and the incorporation of rapid genome sequencing (rGS) in CHD has not been well studied. Though smaller studies demonstrate the value of rGS, they also highlight the burden of results interpretation. We analyze genetic testing in CHD at two time-points, in 2018 and 2022–2023, across a change in clinical testing guidelines from chromosome microarray (CMA) to rGS. Analysis of 421 hospitalized infants with CHD demonstrated consistent genetic testing across time. Overall, after incorporation of rGS in 2022–2023, the diagnostic yield was 6.8% higher compared to 2018, and this pattern was consistent across all patient subtypes analyzed. In 2018, CMA was the most common test performed, with diagnostic results for CHD in 14.3%, while in 2022–2023, rGS was the most frequent test performed, with results diagnostic for CHD in 16.9%. Additionally, rGS identified 44% more unique genetic diagnoses than CMA. This is the largest study to highlight the value of rGS in CHD and has important implications for management.

## INTRODUCTION

Congenital heart disease (CHD) is the most common birth defect and is a leading cause of death in the newborn period.^1–7^ The etiology of CHD is often genetic.^8^ Multiple studies have demonstrated diagnostic utility of genetic testing in patients with CHD,^9–16^ including a recent multicenter cohort analysis of newborns with critical CHD, which confirmed abnormal genetic testing in up to 44% of newborns with CHD.^17^

In 2008, the American Heart Association and the American Academy of Pediatrics recommended genetic testing for patients with CHD and highlighted some of the reasons to obtain a genetic diagnosis.^18^ One of these reasons is that syndromic features are difficult to identify in newborns, therefore genetic testing is often necessary to identify syndromes.^18^ Moreover, genetic testing can identify comorbidity in other organ systems that would benefit from increased screening and identify risk for developmental delay that would benefit from early intervention services. Furthermore, genetic testing may identify risks in family members. Finally, genetic testing increasingly informs prognosis and can guide surgical care and medical management in CHD patients.^19^

Within the past decade, chromosome microarray (CMA) emerged as the preferred genetic testing approach in patients with CHD.^17^ This shift in testing strategy to newer testing modalities, like CMA, can increase genetic diagnoses in newborns with CHD.^20^ Additionally, studies demonstrate CMA can be utilized to identify genotype-phenotype associations in CHD.^21^ However, recently the genetic testing landscape has seen a shift to newer modalities including genome sequencing.

Genome sequencing offers the benefit of identifying structural variation detected by CMA as well as changes at the nucleotide level in a single test.^22^ As costs have decreased and laboratory workflow capacity increased, genomic sequencing has become feasible as a first-line clinical test.^22^ Over 20 recent studies have shown that genome sequencing is useful in critically ill newborns, with higher diagnostic yield and increased clinical utility.^23^ Genome sequencing generates more actionable results and can even reduce the cost of care.^23,24^ While there are limited studies specific to CHD patients, two recent reports utilized rapid genome sequencing (rGS) in CHD patients and showed a high diagnostic yield of 27% and 46%.^25,26^

It is important to note that while genome sequencing has proven valuable, there are limitations. One of the biggest challenges involves the burden of interpreting results. In the recent study “Genomic Medicine in Ill Neonates and Infants,” genomic sequencing led to a high diagnostic yield; however, when up to 67% of results were discordant between labs due to technical differences, variant interpretation differences or both.^27^ These discrepancies highlight the need for further evaluation of comprehensive genome sequencing in CHD.

In 2010, the International Standard Cytogenomic Array Consortium released a consensus statement, recommending CMA analysis as first-tier genetic test for most patients with congenital anomalies, including CHD.^28^ In 2021, the American College of Medical Genetics and Genomics updated genetic testing recommendations for newborns with congenital anomalies, incorporating consideration of exome and genome sequencing as first-tier testing modalities.^29^ Consistent with these international guidelines, in 2014, our center implemented clinical guidelines for universal CMA in all hospitalized infants with CHD. In 2019, we changed the protocol to incorporate molecular-based testing, and in July 2022, we changed our guidelines to universal rGS for all infants with CHD. Here, we analyze genetic testing rate and yield in 2018 and again in 2022–2023, across a shift from CMA to rGS. We show that rGS has diagnostic utility across a range of patient subtypes, comparable to CMA. These findings have important implications for patients with the most common birth defect, CHD.

## MATERIALS/SUBJECTS AND METHODS

### Study Population

We conducted a retrospective analysis of genetic testing practices and results at Riley Hospital for Children at Indiana University Health. To comprehensively identify patients with critical CHD, we utilized the Society of Thoracic Surgeons (STS) National Database. We included children who had surgical repair for CHD at 14 months old or younger between January 1, 2018 and December 31, 2018, as previously described,^20^ and between July 1, 2022 and June 30, 2023. Indiana University Institutional Review Board approved the study (protocol #1408953015) and all research was performed in accordance with relevant ethical guidelines of the 1975 Declaration of Helsinki and all regulations as reflected in approval by the Institutional Review Board. A waiver of informed consent was approved by the Indiana University Institutional Review Board for this minimal risk study. The data was entered into the database deidentified, except for the date of birth, which was used for data integrity. Only summary results are presented within the research article and all data presented within the article is de-identified.

### Data Collection

The patient’s medical records were thoroughly reviewed, and all the study data were entered into a Research Electronic Data Capture (REDCap) database hosted at Indiana University. Additionally, data were obtained directly from the STS National Database. Variables included date of birth, surgery date, sex, race, family history of CHD, presence of other major congenital malformations, history of intrauterine growth restriction (IUGR) or being born small for gestational age (SGA), CHD group, infant of diabetic mother, maternal teratogens (alcohol, illegal drugs, tobacco, and prescription opioids), maternal infection, multiple gestation, gestational age, birth weight, number of cardiac surgeries, deceased status, and extracorporeal membrane oxygenation (ECMO). The medical record was reviewed to identify documentation of additional congenital anomalies, which were further classified by location, including the brain, ear, nose or throat, eye, chest, lung, diaphragm, congenital diaphragmatic hernia, gastrointestinal and abdominal wall, kidney, spleen, pancreas/annular pancreas, liver and gallbladder, anus, imperforate anus or anal atresia, genitourinary, ribs and vertebrae, limbs, lymphatic, lymphatic dysplasia, skin, arteriovenous malformation, and umbilical and categories with too few patients was grouped as “other” during statistical analysis. Participants with at least one congenital anomaly, in addition to CHD, were defined as having multiple congenital anomalies (MCA) to distinguish them from those with isolated CHD.

### Genetic Testing Review

All the available genetic testing results were collected from the medical record, including prenatal cell-free DNA screening (cfDNA), chromosome analysis (karyotype), CMA, fluorescent in situ hybridization analysis (FISH), single-gene sequencing, gene panel sequencing, exome sequencing (ES) and rGS. The clinical testing laboratory report was used for result interpretation. Testing results from the clinical laboratory report were evaluated as “normal,” “pathogenic,” “likely pathogenic,” “variant of unknown significance,” “likely benign,” or “not reported/unknown.” All genetic testing results were independently reviewed by both a genetic counselor and medical geneticist with expertise in cardiovascular genetics and classified as “diagnostic for CHD” versus “not diagnostic for CHD.” Discrepancies were reviewed and resolved by a second medical geneticist with expertise in cardiovascular genetics.

### Statistical Analysis

Univariate comparisons of patients’ characteristics, testing rate, and testing yield were analyzed using Pearson’s Chi-square test (if all expected cell counts in contingency table were ≥ 5) or Fisher’s exact test (if any expected cell counts were < 5) for categorical variables, and one-way analysis of variance for continuous variables. All analyses were conducted in R version 4.0.2 (The R Foundation for Statistical Computing, Vienna, Austria).

## RESULTS

### Characteristics of the Cohort

The study included 421 CHD patients, among which 308 had isolated CHD and 113 had CHD + MCA (Table 1, Supplemental Table 1). More than half of the patients (57%) were male, and the majority (81.7%) were white. Among the total cohort, 78.4% were born full term and 13.5% were IUGR or SGA. Additionally, 13.1% of the patients had mothers with diabetes, 11.6% were exposed to maternal teratogens (such as alcohol, tobacco, or illicit drugs), and 24.0% were exposed to maternal infection. In the cohort, patients required an average of 1.6 cardiac surgeries, and 11.6% of the total cohort needed ECMO. At the time of data collection, 8.8% of the patients were deceased (Table 1).

In 2014, our institution implemented guidelines that recommended CMA for most infants hospitalized with CHD; in 2022, we updated guidelines to recommend rGS as a first-tier testing modality. We analyzed a time period in 2018, where CMA was recommended, and 2022–2023, where rGS was the first-tier testing modality. There were no significant differences between the two time periods by characteristics of: sex, race, maternal diabetes, maternal infection, fetal growth restriction, ECMO, or vitality status. However, there was a significantly higher rate of family CHD history, specifically as ascertained by a genetics provider in 2022–2023, and a higher frequency of congenital anomalies identified in 2022–2023, possibly both reflecting the higher rate of medical genetics involvement in 2022–2023 (Tables 1 and 2). There was a smaller number of preterm births and a slightly larger number of late preterm births in 2022–2023, compared to 2018. Overall, the patient characteristics were similar between the 2018 and 2022–2023 cohort.

### Genetic Testing Rate

Genetic testing was conducted in 327 out of 421 patients during the two time periods, which accounts for 77.7% of the entire cohort (Table 2). Out of the 308 patients with isolated CHD, 226 (73.4%) underwent genetic testing. In comparison, those with CHD+MCA underwent testing more frequently, with 101 out of 113 patients (89.4%) being tested (Table 2).

In 2018, 143 out of 190 patients (75.3%) underwent testing, and in 2022–2023, 184 out of 231 patients (79.7%) were tested (p=0.338). Although the overall testing rate was slightly higher in 2022–2023 compared to 2018, the difference between these time periods was not statistically significant (Table 2). This trend was observed across all subtypes of patients, including those with isolated CHD, with 114 out of 157 (72.6%) patients tested in 2018 and 112 out of 151 (74.2%) in 2022–2023 (p=0.856) (Table 2). Similarly, among patients with CHD+MCA, the number of patients tested was slightly higher but not significantly different, with 29 out of 33 (87.9%) tested in 2018 and 72 out of 80 (90.0%) in 2022–2023 (p=0.744) (Table 2).

### Genetic Testing Results

Next, we analyzed genetic testing results and initially segregated the results into two categories, normal versus abnormal. We referred all abnormal results to an expert in cardiovascular genetics for further categorization as diagnostic for CHD (Table 2). Out of the 327 total patients who underwent genetic testing, 88 patients (26.9%) had diagnostic results (Table 2). Among the 226 tested patients with isolated CHD, 51 patients (22.6%) had diagnostic results while a higher number of patients with CHD+MCA had diagnostic results, including 37 out of 101 (36.6%) tested patients (Table 2).

The diagnostic yield was 6.8% higher in 2022–2023 compared to 2018. In 2018, out of 143 tested patients, 33 (23.1%) had diagnostic testing, while in 2022–2023, out of 184 tested patients, 55 (29.9%) had diagnostic results, (though the difference did not meet statistical significance (p=0.210)) (Table 2).

This pattern was consistent across all patient subtypes. In those with isolated CHD, the diagnostic yield was 3% higher in 2022–2023 compared to 2018, with 24 diagnostic results from 114 tested patients (21.1%) in 2018; while in 2022–2023, out of 112 tested patients, 27 had diagnostic results (24.1%) (p=0.696) (Table 2). Similarly, in patients with CHD+MCA, the diagnostic yield was 7.9% higher in 2022–2023 compared to 2018, where there were 9 out of 29 (31.0%) diagnostic results in 2018 compared to 28 out of 72 (38.9%) in 2022–2023 (p=0.608) (Table 2).

Overall, the diagnostic yield was 6.8% higher in 2022–2023 compared to 2018, after the incorporation of rGS, and this pattern was consistent across patient subtypes analyzed.

### Medical Genetic Involvement

In 2014, new guidelines were implemented at our site that recommended medical genetics evaluation for all patients with CHD to assist in genetic testing. Out of 421 patients, medical genetics was involved with 288 patients (68.4%) (Table 2). The frequency of medical genetics involvement increased between the two study periods, with 121 of 190 patients (63.7%) being evaluated in 2018 compared to 167 of 231 (72.3%) in 2022–2023. However, at 5% level this was not statistically significant (p=0.074) (Table 2). Additionally, the reported family history of CHD also increased from 2018 to 2022–2023. This could be due to an increase in medical history taking, which may be a result of the rise in medical genetics involvement.

### Genetic Testing Rate by Testing Modality

We conducted a more in-depth analysis of the individual testing modalities. The most frequently used genetic test overall was CMA, which was performed on 167 of 421 patients (39.7%), followed by rGS, performed in 90 patients (21.4%) (Table 3). The choice of testing modality reflected the institutional guideline recommendation in each time period. In 2018, CMA was the most frequent test, used in 126 of 190 patients (66.3%); whereas in 2022–2023, rGS was the most frequent test, completed in 89 of 231 (38.5%) (Table 3).

Although the overall testing rate did not significantly change from 2018 to 2022–2023 (Table 2), there was a significant difference between the individual testing modalities, reflecting a shift in testing between the two periods (Table 3). The use of CMA decreased significantly in 2022–2023, from 66.3% to 17.7% (p<0.001) (Table 3). Meanwhile, there was a significant increase in rGS, from only 0.5% in 2018 to 38.5% in 2022–2023 (p<0.001) (Table 3).

Similarly, there was an increase in the use of cfDNA, from 5 of 190 (2.6%) in 2018, to 43 of 231 (18.6%) in 2022–2023 (p<0.001), and in gene panels, from 7 of 190 (3.7%) in 2018 to 40 of 231 (17.3%) in 2022–2023 (p<0.001) (Table 3). The use of chromosome analysis remained consistent, occurring in 23 of 190 in 2018 (12.1%) and 32 of 231 in 2022–2023 (13.9%) (Table 3). FISH and single gene testing were rare in both time periods.

There was a trend of increased ES over time, from 6 of 190 (3.2%) in 2018 to 18 of 231 (7.8%) in 2022–2023 (p=0.067); however, the trend did not meet statistical significance at 5% level (Table 3). This trend reflects clinical guidelines, which recommended ES for a two-month period, immediately prior to the update in 2022–2023, for universal rGS in all infants with CHD.

Overall, the results demonstrate a significant shift in testing modalities, from a predominance of CMA in 2018 to rGS in 2022–2023.

### Genetic Testing Results by Modality

As described previously, a medical geneticist with expertise in cardiovascular genetics analyzed the results of all genetic testing and determined whether each finding was diagnostic for CHD, and the overall diagnostic yield was 26.9% (Table 2), whereas the yield of the individual testing modalities ranged from 6.4% to 63.6% (Table 4). The most commonly performed tests were CMA and rGS. CMA had diagnostic results in 28 of 167 patients (16.8%), and rGS had diagnostic results in 15 of 90 (16.7%), overall. As described, there was a significant shift in use from CMA in 2018 to rGS in 2022–2023. CMA was the most common modality in 2018, used in 126 patients, and 18 patients (14.3%) had diagnostic results. The most frequent test performed in 2023–2023 was rGS, performed in 89 patients, with diagnostic results in 15 (16.9%) (Table 4).

We observed a significant change in the testing rate for multiple testing modalities between 2018 and 2022–2023; however, only chromosome analysis and cfDNA demonstrated a significant change in testing yield.

In 2018, 10 of 23 chromosome analysis results were diagnostic (43.5%), while in 2022–2023, there were 25 of 32 diagnostic results (78.1%), representing a significant increase (p=0.019)(Table 4). Targeted chromosome analysis was used in suspected aneuploidies, and indeed, all abnormal chromosome analysis results in the cohort represent a diagnosis of Trisomy 21, aside from two Turner Syndrome diagnoses and one larger deletion. The significant increase in yield in 2022–2023 likely reflects a better-targeted use of chromosome analysis for suspected aneuploidies in the rGS era.

In 2018, gene panel testing was used in 7 patients without any diagnostic findings (0%). In 2022–2023, gene panel testing was performed on 40 patients, and diagnostic findings were observed in 3 patients (7.5%) (Table 4).

ES showed a trend toward increased use, with a consistent yield, although the numbers of patients were small. In 2018, 6 patients were tested, and diagnostic findings were observed in 2 (33%). In 2022–2023,18 patients were tested, and diagnostic findings were observed in 7 (38.9%).

Overall, the results demonstrate that the incorporation of rGS increased the diagnostic yield of genetic testing (6.8% increase in diagnostic yield in 2022–2023)(Table 2), and rGS had a higher diagnostic yield when used as a first-line modality in CHD (yield of CMA 14.3% in 2018 and yield of rGS 16.9% in 2022–2023)(Table 4).

### CMA and rGS Detailed Results

According to the findings, the inclusion of rGS improved the yield of genetic testing, resulting in a 6.8% increase in diagnostic yield between 2018 and 2022–2023 (Table 2). Additionally, when used as the first line of testing, rGS exhibited a higher diagnostic yield at the patient level, (16.9% yield of rGS in 2022–2023 vs 14.3% for CMA in 2018 (Table 4)). However, each patient often had multiple abnormal findings reported. Therefore, next, we analyzed CMA and rGS at the level of testing results breakdown when both time period results were grouped (Supplemental Table 2) (in contrast to the results per patient in Table 4.) Among the CMA results, 62.6% were negative or normal, while in the case of rGS, this figure was 74.1%. 15.9% of CMA results were pathogenic variants compared to 12.0% of rGS results. Likely pathogenic results represented 2.7% of CMA results and 1.3% of rGS results. Variants of uncertain significance made up 14.8% of CMA results and 12% of rGS results. Overall, the results breakdown between CMA and rGS were mostly consistent. However, there was a slightly increased proportion of abnormal results in CMA (Supplemental Table 2); in the setting of increased diagnostic results of rGS (Tables 2–4), this indicates CMA may have a higher number of nondiagnostic findings compared to rGS.

All diagnostic genetic testing results from the two most common modalities, CMA and rGS are provided (Table 5). In total, more than 20 unique genetic findings were identified through both CMA and rGS, with rGS detecting 44% more unique genetic diagnoses compared to CMA (13 versus 9) (Table 5).

## Discussion

This study is the largest to date evaluating a clinical program for rGS in infants with CHD. Our findings demonstrate that rGS is a useful tool in infants with CHD, comparable or slightly improved when compared to modalities such as CMA, with a higher diagnostic yield and a more diverse spectrum of genetic diagnoses identified. The results of this study have significant implications for the management of infants with CHD, the most common birth defect.

Our study demonstrates the benefit of genetic testing in patients with CHD, across multiple patient subtypes and genetic testing modalities. The diagnostic yield of genetic testing across both time periods analyzed was found to be 26.9% (Table 2), which is consistent with the genetic contribution to CHD reported in epidemiological studies and similar to other studies of genetic testing in infants with CHD, which report yields 25%−50%, depending on ascertainment protocols, often reflecting clinical program and referral sources at each institution.^11,13,15^ Our yield is on the lower end because it represents only results confirmed clearly diagnostic for CHD after a secondary review. It is possible some of the excluded diagnoses will ultimately be found to be causative of CHD as well. Our study period represents an early phase of adoption of rGS testing, and it is possible that with longer rGS usage, we may see the diagnostic yield increase. Nonetheless, genetic testing showed high yields across almost every patient subtype, including both isolated CHD (22.6%) and patients with CHD+MCA (36.6%).

Our study examines two distinct time periods, in 2018 and 2022–2023. At our institution, we updated testing guidelines to recommend rGS in mid-2022. As a result, there was a significant shift from CMA being the most common test in 2018 to rGS being the most common test in 2022–2023 (Table 3). Our institution follows guidelines for testing patients with congenital anomalies, which have evolved over time. Our guideline for CMA was implemented based on ISCA guidelines in 2014 and was in place from 2014 until 2018. It was updated in December of 2019 to an exome-based gene panel plus CMA. Based on the American College of Medical Genetics’ 2021 guidelines for exome and genome sequencing in newborns with birth defects, we updated our guidelines in 2021 to Exome Sequencing (ES), and finally, in 2022 to rGS. The time period of 2022–2023 reflects a relatively new recommendation and a diverse patient population, which may represent protocol compliance at various stages. Nevertheless, rGS is the most commonly used test during this period, indicating a good uptake of the new protocol. Genetic testing protocols have shown value in the CHD population, including reducing costs and increasing genetic diagnoses. In a separate study, we demonstrated that genetic testing guidelines increased testing rates and shifted testing towards newer modalities, with the potential to identify increased genetic diagnoses and impact patient care.^11,20^

In this study, we found that rGS is as useful as or slightly better than CMA in detecting genetic abnormalities in infants with CHD. After the incorporation of rGS in 2022–2023, the overall diagnostic yield of genetic testing increased by 6.8% (Table 2). In directly comparing modalities, CMA had a diagnostic rate of 14.3% for CHD when used as a first-tier diagnostic tool in 2018, whereas rGS had a diagnostic rate of 16.9% for CHD in 2022–2023 (Table 4). Furthermore, rGS identified 44% more unique genetic diagnoses when compared to CMA (Table 5).

These results are consistent with recent studies of genome sequencing in critically ill infants. One study found that in patients referred for genetics evaluation, genome sequencing had a yield of 41% in CHD, compared to 24% for CMA.^30^ Another study found that when compared directly to CMA or CMA with a gene panel, genome sequencing had a higher yield (8% and 13% versus 34%).^31^ There have also been recent reports of rGS specifically in CHD. One study of a small, selected cohort of 24 CHD patients found that rGS improved patient care and reduced costs, with a diagnostic yield of up to 46%.^32^ Another study of a slightly larger population of 48 patients had a diagnostic yield of 27%.^25^ Although diagnostic yield can be a flawed metric since it is dependent on patient ascertainment and can be elevated in highly selected cohorts, there is clear value in rGS in CHD patients. Our approach to hospitalized infants with CHD is to test comprehensively and 77.7% underwent genetic testing. Our data from 2022–2023 captures our change in protocol to rGS and our cohort of 90 patients tested confirms that rGS is a useful diagnostic tool for infants with CHD.

Genome sequencing has immense potential as it provides a broader capture of genetic anomalies. Studies have shown that the laboratory workflow for genome sequencing can be simpler, with improving cost and diagnostic rates, making it a first-tier genetic test.^24^ However, there are limitations to rGS, as previous studies have noted major discrepancies in variant interpretation that can impact its utilization.^27^ Nevertheless, our data showed that rGS may have had fewer nondiagnostic findings, including fewer VUS compared to CMA (12% versus 14.8%, Supplemental Table 2.) Our data also revealed an increased rate of medical genetics involvement in 2022–2023, accompanying the shift to rGS, which may have helped overcome this limitation.

The increased utilization of genetics providers in the 2022–2023 cohort likely aided the interpretation of results. There were other differences between our 2018 and 2022–2023 cohorts, attributable to increased medical genetic involvement in 2022–2023, including a significantly higher rate of family CHD history ascertained by a genetics provider and a higher frequency of congenital anomalies identified in 2022–2023 (Table 1). Our institutional guidelines recommend consultation with medical genetics in all CHD patients; however, medical genetics involvement was not universal, occurring in 68.4% (Table 2). With the increasing complexity of genetic testing results and the changing testing recommendations, the involvement of medical geneticists is becoming increasingly important. Medical genetics can help with testing strategies and interpreting results, and multiple studies have shown that medical genetics can increase testing utilization and yield, as well as improve care.^11^ Although there is a shortage of genetics expertise in the workforce, we expect telehealth to provide an opportunity for increased involvement in the future. Additionally, we expect that the identification of emerging genes attributable to CHD and advanced technology in variant interpretation will likely continue to improve the diagnostic yield of genetic testing.

Our protocol recommended universal genetic testing in patients with CHD; however, in our cohort, the genetic testing rate was 77.7%, indicating screening was not universal and there was a lack of strict compliance with the recommended protocol. Single center studies indicate genetic testing is underutilized and highly variable^9–16^ and our recent multicenter evaluation of newborns with CHD indicated genetic testing rates of 42–78% across 5 centers.^17^ Identifying the genetic cause of CHD has been shown to improve patient care by providing information on prognosis, identifying other organ system involvement, and allowing for the implementation of health supervision and early intervention. Moreover, identifying a genetic origin enables the assessment of recurrence risk for patients as well as the risk to other family members.^10,18,33,34^ Therefore, quality improvement efforts are necessary to ensure that guidelines for genetic testing are being followed.

We should note that our study has some limitations. First, we only used genetic testing results that were available within the electronic medical record, which means that we did not include any testing that may have been carried out at outside hospitals or outside the period of data collection. Moreover, our study only includes patients who underwent surgery soon after birth and, therefore, excludes less critically ill infants. On the other hand, it may not be representative of the most critically ill infants who died before surgery and who are at high risk of genetic diagnoses. To improve our understanding of this issue, future studies should aim to increase sample sizes and involve multiple institutions.

Although our study reports the diagnostic yield, it does not measure clinical utility. Clinical utility refers to the ability of genome sequencing to change medical or surgical management based on its results, and this level of detail is not captured in our data. Studies have shown the clinical usefulness of genome sequencing in critically ill infants from small populations.^35^ Future studies should expand these efforts.

## Conclusions

Overall, this is the largest study highlighting the use of rGS in infants with CHD. We also demonstrate that overall, genetic testing is useful in infants with CHD, across a range of patient subtypes. We show that rGS has utility comparable or slightly improved to older modalities like CMA in infants with CHD. The findings have important implications for the evaluation of CHD patients.

## Figures and Tables

**Table 1.** Patient characteristics

	Total	2018	2022–2023	*p*-value
	(N=421)	(N=190)	(N=231)	
**Sex**				0.720
Male	240 (57.0%)	106 (55.8%)	134 (58.0%)	
Female	181 (43.0%)	84 (44.2%)	97 (42.0%)	
**Race**				0.668
Caucasian	344 (81.7%)	154 (81.1%)	190 (82.3%)	
Black/African American	58 (13.8%)	26 (13.7%)	32 (13.9%)	
Other	18 (4.3%)	10 (5.3%)	8 (3.5%)	
Unknown	1 (0.2%)	0 (0.0%)	1 (0.4%)	
**Family History of CHD - Genetics Provider Only**				0.003
Yes	87 (20.7%)	24 (12.6%)	63 (27.3%)	
No	198 (47.0%)	94 (49.5%)	104 (45.0%)	
Unknown	136 (32.3%)	72 (37.9%)	64 (27.7%)	
**Family History of CHD - Other Specialty (Non-genetics Provider(s))**				0.170
Yes	35 (8.3%)	7 (3.7%)	28 (12.1%)	
No	297 (70.5%)	98 (51.6%)	199 (86.1%)	
Unknown	89 (21.1%)	85 (44.7%)	4 (1.7%)	
**Multiple Congenital Anomalies**				<0.001
Yes	113 (26.8%)	33 (17.4%)	80 (34.6%)	
No	308 (73.2%)	157 (82.6%)	151 (65.4%)	
**Infant of a Diabetic Mother**				>0.999
Yes	55 (13.1%)	25 (13.2%)	30 (13.0%)	
No	276 (65.6%)	127 (66.8%)	149 (64.5%)	
Unknown	90 (21.4%)	38 (20.0%)	52 (22.5%)	
**Maternal Teratogens**				0.053
Yes	49 (11.6%)	16 (8.4%)	33 (14.3%)	
No	283 (67.2%)	138 (72.6%)	145 (62.8%)	
Unknown	89 (21.1%)	36 (18.9%)	53 (22.9%)	
**Maternal Infection**				0.213
Yes	101 (24.0%)	40 (21.1%)	61 (26.4%)	
No	237 (56.3%)	113 (59.5%)	124 (53.7%)	
Unknown	83 (19.7%)	37 (19.5%)	46 (19.9%)	
**Average Weight at Birth (kg)**				0.078
Mean (SD)	2.91 (0.76)	2.83 (0.86)	2.97 (0.66)	
**Gestational Age**				0.004
Term (>= 37 wk)	330 (78.4%)	146 (76.8%)	184 (79.7%)	
Late Preterm (32 – 37 wk)	67 (15.9%)	28 (14.7%)	39 (16.9%)	
Preterm (<32 wk)	18 (4.3%)	15 (7.9%)	3 (1.3%)	
Unknown	6 (1.4%)	1 (0.5%)	5 (2.2%)	
**History of IUGR or SGA**				0.777
Yes	57 (13.5%)	28 (14.7%)	29 (12.6%)	
No	289 (68.6%)	133 (70.0%)	156 (67.5%)	
Unknown	75 (17.8%)	29 (15.3%)	46 (19.9%)	
**Number of Cardiac Surgeries**				0.001
Mean (SD)	1.6 (0.95)	1.78 (1.14)	1.46 (0.73)	
**ECMO Required**				0.466
Yes	49 (11.6%)	25 (13.2%)	24 (10.4%)	
No	372 (88.4%)	165 (86.8%)	207 (89.6%)	
**Deceased**				0.782
Yes	37 (8.8%)	18 (9.5%)	19 (8.2%)	
Unknown	384 (91.2%)	172 (90.5%)	212 (91.8%)	

Abbreviations: CHD, congenital heart disease; MCA, multiple congenital anomalies IUGR, SGA, ECMO

**Table 2:** Genetic testing rate, diagnostic yield and medical genetics involvement

			Total	2018	2022–2023	*p*-value
**All Patients**						
	Testing	N	421	190	231	0.338
		Yes	327 (77.7%)	143 (75.3%)	184 (79.7%)	
		No	94 (22.3%)	47 (24.7%)	47 (20.3%)	
	Testing Diagnostic for CHD	N	327	143	184	0.210
		Yes	88 (26.9%)	33 (23.1%)	55 (29.9%)	
		No	239 (73.1%)	110 (76.9%)	129 (70.1%)	
	Medical Genetics Involvement	N	421	190	231	0.074
		Yes	288 (68.4%)	121 (63.7%)	167 (72.3%)	
		No	133 (31.6%)	69 (36.3%)	64 (27.7%)	
**Isolated CHD**						
	Testing	N	308	157	151	0.856
		Yes	226 (73.4%)	114 (72.6%)	112 (74.2%)	
		No	82 (26.6%)	43 (27.4%)	39 (25.8%)	
	Testing Diagnostic for CHD	N	226	114	112	0.696
		Yes	51 (22.6%)	24 (21.1%)	27 (24.1%)	
		No	175 (77.4%)	90 (78.9%)	85 (75.9%)	
	Medical Genetics Involvement	N	308	157	151	0.160
		Yes	197 (64.0%)	94 (59.9%)	103 (68.2%)	
		No	111 (36.0%)	63 (40.1%)	48 (31.8%)	
**CHD + MCA**						
	Testing	N	113	33	80	0.744
		Yes	101 (89.4%)	29 (87.9%)	72 (90.0%)	
		No	12 (10.6%)	4 (12.1%)	8 (10.0%)	
	Testing Diagnostic for CHD	N	101	29	72	0.608
		Yes	37 (36.6%)	9 (31.0%)	28 (38.9%)	
		No	64 (63.4%)	20 (69.0%)	44 (61.1%)	
	Medical Genetics Involvement	N	113	33	80	>0.999
		Yes	91 (80.5%)	27 (81.8%)	64 (80.0%)	
		No	22 (19.5%)	6 (18.2%)	16 (20.0%)	

Abbreviations: CHD, congenital heart disease; MCA, multiple congenital anomalies

**Table 3:** Testing rate by genetic testing modality

		Total	2018	2022–2023	*p*-value
		(N = 421)	(N = 190)	(N = 231)	
**cfDNA**					<0.001
	Yes	48 (11.4%)	5 (2.6%)	43 (18.6%)	
	No	373 (88.6%)	185 (97.4%)	188 (81.4%)	
**Chromosome Analysis**					0.701
	Yes	55 (13.1%)	23 (12.1%)	32 (13.9%)	
	No	366 (86.9%)	167 (87.9%)	199 (86.1%)	
**FISH**					0.476
	Yes	8 (1.9%)	5 (2.6%)	3 (1.3%)	
	No	413 (98.1%)	185 (97.4%)	228 (98.7%)	
**CMA**					<0.001
	Yes	167 (39.7%)	126 (66.3%)	41 (17.7%)	
	No	254 (60.3%)	64 (33.7%)	190 (82.3%)	
**Single Gene Testing**					>0.999
	Yes	2 (0.5%)	1 (0.5%)	1 (0.4%)	
	No	419 (99.5%)	189 (99.5%)	230 (99.6%)	
**Panel**					<0.001
	Yes	47 (11.2%)	7 (3.7%)	40 (17.3%)	
	No	374 (88.8%)	183 (96.3%)	191 (82.7%)	
**Exome Sequencing**					0.067
	Yes	24 (5.7%)	6 (3.2%)	18 (7.8%)	
	No	397 (94.3%)	184 (96.8%)	213 (92.2%)	
**Rapid Genome Sequencing**					<0.001
	Yes	90 (21.4%)	1 (0.5%)	89 (38.5%)	
	No	331 (78.6%)	189 (99.5%)	142 (61.5%)	
**Other**					0.706
	Yes	7 (1.7%)	4 (2.1%)	3 (1.3%)	
	No	414 (98.3%)	186 (97.9%)	228 (98.7%)	

Abbreviations: cfDNA, cell-free DNA; CMA, chromosome microarray; FISH, fluorescence in situ hybridization

**Table 4:** Testing yield by genetic testing modality in 2018 and 2022–2023

		Total	2018	2022–2023	*p*-value
**cfDNA**	N	48	5	43	0.049
	Yes	4 (8.3%)	2 (40.0%)	2 (4.7%)	
	No	44 (91.7%)	3 (60.0%)	41 (95.3%)	
**Chromosome Analysis**	N	55	23	32	0.019
	Yes	35 (63.6%)	10 (43.5%)	25 (78.1%)	
	No	20 (36.4%)	13 (56.5%)	7 (21.9%)	
**FISH**	N	8	5	3	>0.999
	Yes	1 (12.5%)	1 (20.0%)	0 (0.0%)	
	No	7 (87.5%)	4 (80.0%)	3 (100.0%)	
**CMA**	N	167	126	41	0.206
	Yes	28 (16.8%)	18 (14.3%)	10 (24.4%)	
	No	139 (83.2%)	108 (85.7%)	31 (75.6%)	
**Single Gene Testing**	N	2	1	1	>0.999
	Yes	1 (50.0%)	1 (100.0%)	0 (0.0%)	
	No	1 (50.0%)	0 (0.0%)	1 (100.0%)	
**Gene Panel**	N	47	7	40	>0.999
	Yes	3 (6.4%)	0 (0.0%)	3 (7.5%)	
	No	44 (93.6%)	7 (100.0%)	37 (92.5%)	
**Exome Sequencing**	N	24	6	18	>0.999
	Yes	9 (37.5%)	2 (33.3%)	7 (38.9%)	
	No	15 (62.5%)	4 (66.7%)	11 (61.1%)	
**Rapid Genome Sequencing**	N	90	1	89	>0.999
	Yes	15 (16.7%)	0 (0.0%)	15 (16.9%)	
	No	75 (83.3%)	1 (100.0%)	74 (83.1%)	
**Other**	N	7	4	3	>0.999
	Yes	1 (14.3%)	1 (25.0%)	0 (0.0%)	
	No	6 (85.7%)	3 (75.0%)	3 (100.0%)	

* The term “yield” for cfDNA refers to a positive screening result, which generally requires some confirmatory genetic test via prenatal amniocentesis or postnatally; therefore, it only represents abnormal results, whereas other modalities were reviewed and determined by an expert and determined diagnostic for CHD.

Abbreviations: cfDNA, cell-free DNA; CMA, chromosome microarray; FISH, fluorescence in situ hybridization

**Table 5:** Findings diagnostic for CHD by CMA and rGS

	CMA	rGS
22q11.21 deletion	15	3*
Trisomy 21	6	2
Beckwith-Wiedemann Syndrome	2	
Turner Syndrome	2	
Trisomy 9, mosaic	1	
14q24.3q32.12 deletion	1	
16p13.11-p12.3 duplication	1	
8p23.1 duplication	1	
10q23.22q26.3 duplication	1	
*DNAH5* variant		2
*GATA6* variant		1
*NOTCH1* variant		1
*SETD5* variant		1
*TBX1* variant		1
*TRRAP* variant		1
1q21.1 duplication		1
1q21.1 deletion		1
12P13.33p13.32 deletion		1
12q224.33 deletion		1
17p13.3 microduplication		1

Abbreviations: CHD, Congenital heart disease; CMA, chromosome microarray; rGS, rapid genome sequencing

* rGS performed due to additional medical concerns. Results reconfirmed Trisomy 21.

## Data Availability

De-identified datasets are available upon reasonable request to the corresponding author.
